# Mutations in *LMNA* Modulate the Lamin A - Nesprin-2 Interaction and Cause LINC Complex Alterations

**DOI:** 10.1371/journal.pone.0071850

**Published:** 2013-08-20

**Authors:** Liu Yang, Martina Munck, Karthic Swaminathan, Larisa E. Kapinos, Angelika A. Noegel, Sascha Neumann

**Affiliations:** 1 Institute for Biochemistry I, Medical Faculty, University of Cologne, and Center for Molecular Medicine Cologne (CMMC) and Cologne Cluster on Cellular Stress Responses in Aging-Associated Diseases (CECAD), Medical Faculty, University of Cologne, Cologne, Germany; 2 Biozentrum and the Nanoscience Institute, University of Basel, Basel, Switzerland; University of Florida, United States of America

## Abstract

**Background:**

In eukaryotes the genetic material is enclosed by a continuous membrane system, the nuclear envelope (NE). Along the NE specific proteins assemble to form meshworks and mutations in these proteins have been described in a group of human diseases called laminopathies. Laminopathies include lipodystrophies, muscle and cardiac diseases as well as metabolic or progeroid syndromes. Most laminopathies are caused by mutations in the *LMNA*gene encoding lamins A/C. Together with Nesprins (**N**uclear **E**nvelope **Sp**ectrin **R**epeat Prote**ins**) they are core components of the LINC complex (**Li**nker of **N**ucleoskeleton and **C**ytoskeleton). The LINC complex connects the nucleoskeleton and the cytoskeleton and plays a role in the transfer of mechanically induced signals along the NE into the nucleus, and its components have been attributed functions in maintaining nuclear and cellular organization as well as signal transduction.

**Results:**

Here we narrowed down the interaction sites between lamin A and Nesprin-2 to aa 403–425 in lamin A and aa 6146–6347 in Nesprin-2. Laminopathic mutations in and around the involved region of lamin A (R401C, G411D, G413C, V415I, R419C, L421P, R427G, Q432X) modulate the interaction with Nesprin-2 and this may contribute to the disease phenotype. The most notable mutation is the lamin A mutation Q432X that alters LINC complex protein assemblies and causes chromosomal and transcription factor rearrangements.

**Conclusion:**

Mutations in Nesprin-2 and lamin A are characterised by complex genotype phenotype relations. Our data show that each mutation in *LMNA*analysed here has a distinct impact on the interaction among both proteins that substantially explains how distinct mutations in widely expressed genes lead to the formation of phenotypically different diseases.

## Introduction

A hallmark of eukaryotes is the compartmentalization of the cell and the presence of organelles. Among them, the largest one is the nucleus that harbors the genetic material. The nucleus is surrounded by a continuous membrane system, the nuclear envelope (NE), that consists of two concentric membranes, inner (INM) and outer (ONM) nuclear membrane. They are separated by the perinuclear space and integrated along the nuclear pore complexes. The ONM is continuous with the endoplasmic reticulum (ER). Membrane invaginations that might consist of one or both nuclear membranes can reach into the nucleus to form the nucleoplasmic reticulum [1]. Research in the past decade revealed the existence of large macromolecular protein complexes present along or across the nuclear membranes [2]–[6]. The biological importance of these assemblies is becoming more and more evident since the number of human diseases that are known to arise from mutations in their components is continuously increasing. However, the majority is due to mutations in lamin A/C proteins which are encoded by the *LMNA* gene.

Lamins are type V intermediate filament proteins that form a meshwork underlying the INM. They are grouped into A- and B-type lamins. The latter ones are encoded by the *LMNB1* (lamin B1) and *LMNB2* (lamin B2) gene [7]. The *LMNA* gene encodes four A-type lamins (A, AD10, C and C2) that are generated by alternative splicing and posttranslational modifications. Lamins A and C represent the major isoforms. The structure of lamins follows a tripartide organization into an N-terminal head, a central rod domain and a C-terminal tail. Lamins assemble by forming parallel head to tail dimers stabilized by coiled coil structures in the central rod domain. Dimers then assemble into a head to tail polymer. Higher order structures are achieved by anti-parallel, lateral polymer assemblies, the protofilaments [8]. Three to four protofilaments assemble to intermediate filaments with a diameter of about 10 nm [9].

So far more than 400 disease causing mutations have been reported spread along the entire *LMNA*gene [9], [10]. These mutations cause several distinct diseases that include forms of muscular dystrophy and cardiomyopathy, metabolic syndrome and lipodystrophy, neuropathies and progeroid syndromes. This is surprising as lamin A/C proteins are ubiquitously expressed proteins and points towards tissue or development specific interactions [9].

Currently three mutually non-exclusive hypotheses exist to explain how mutations in nuclear envelope proteins might result in the formation of laminopathies [10]. The first one is the structural hypothesis that refers to the role of LINC complex components in maintaining nuclear architecture and mechanotransduction across the nuclear envelope. Mutations in LINC complex components might cause weakened or strengthened interactions that lead to misshapen nuclei, a hallmark of laminopathies, and impaired transfer of mechanically induced signals across the NE [11], [12]. The second hypothesis explains the role of NE proteins in the formation in laminopathies by their ability to regulate signaling events in a way distinct from mechanotransduction. Various NE proteins have been shown to interact, sequester and regulate transcription factor accessibility to the nucleus and chromatin and therefore control the gene expression profile of a cell [13], [14]. The third hypothesis explains the formation of laminopathies by the accumulation of mutated lamin proteins and the accompanied cell toxic gain of function of these aggregates [15].

Along the nuclear envelope lamins A/C interact with integral proteins of the NE like SUN-1/-2, Emerin, with transcription factors like Fos, SREBP1 and with organizers of chromatin organization like histones or the cell cycle regulator Cyclin D3 [16]–[21]. Additionally, lamins A/C interact with Nesprin-2 [22]. Nesprins (**N**uclear **E**nvelope **Sp**ectrin **R**epeat Prote**ins**) are proteins that mainly reside along the INM and ONM. So far four Nesprins have been described in mammals (Nesprin-1, -2, -3 and -4). Each is encoded by a single gene that gives rise to multiple isoforms. The number of identified isoforms is still increasing. So far 21 isoforms have been identified for Nesprin-1 and 14 for Nesprin-2 [23], [24]. For Nesprin-3 two variants are known and one for the epithelial specific Nesprin-4 [25], [26]. Nesprins broadly differ in their molecular masses from the smaller Nesprin-3 (∼100 kDa) and 4 (∼40 kDa) to the biggest, the so called giant isoforms of Nesprin-1 and -2 that reach up to 1 MDa or 800 kDa, respectively. Structural characteristics that are shared by most Nesprin isoforms are the C-terminal KASH (**K**larsicht/**A**NC-1/**S**yne **h**omology) domain that is a transmembrane domain and spectrin repeats that form the basis of the central rod segment. Even the largest Nesprins contain only one transmembrane segment that is sufficient for connecting the proteins with the membrane. The KASH domain additionally contains a consensus motif at the C-terminus for the interaction with integral proteins of the INM, the SUN (Sad1/UNC-84) proteins. The interaction of the KASH domain of the Nesprins and the SUN domain of the SUN proteins in the PNS forms the core of the LINC complex. The LINC complex is a protein complex that traverses the nuclear envelope for the direct and mechanic coupling of nucleoplasmic and cytoplasmic components [6]. At their N-termini the different Nesprins contain binding sites that mediate direct or indirect connections to the actin filament system, microtubules or the intermediate filament system and therefore an integration of the nucleus into the cytoskeleton of cells.

Knowledge about nuclear envelope proteins and their involvement in the formation of large macromolecular protein complexes along the nuclear envelope is continuously increasing. However, the exact nature of the interaction sites between certain LINC complex components and the effect of distinct mutations for particular interactions are still incomplete. A detailed understanding of protein interactions will help to understand the pathological role of disease causing mutations. In the present study we have narrowed down the interaction sites between lamin A/C and Nesprin-2 and explored the impact of eight laminopathic *LMNA* mutations [R401C (1201C>T) [9], [27], [28], G411D (1232G>A) [29], G413C (1237G>T) (www.umd.be/LMNA), V415I (1243G>A) [9], [30], R419C (1255C>T) [31], L421P (1262T>C) [32], [33], R427G (1279C>G) (www.umd.be/LMNA), Q432X (1294C>T) [9], [34]] on the binding capacities of Nesprin-2 for lamin A/C and the formation of LINC complex assemblies. Mutations in lamin A/C affected the interaction with Nesprin-2 in a spectrum from increasing to decreasing. However most of the described mutations had no obvious effect on the localization of NE proteins along the NE. An exception was the truncation mutation Q432X that caused severe aggregate formations of the mutated lamin A/C protein and the sequestration of nuclear envelope proteins.

## Materials and Methods

### Plasmids and site directed mutagenesis

The amino acid positions of GST and GFP Nesprin-2 proteins used in this study refer to Nesprin-2 giant and have been described elsewhere [14], [22]. The lamin A fragments 1–263, 264–402, 345–425 cloned into pPET-TEV expression vector are described in [35]. The Lamin A sequence 436–548 is inserted into pET24d (Novagen). WT lamin A amino acids 403–425 were cloned into pEGFP-C2 (Clontech) via *EcoRI* and *BamHI* restriction sites and the following primers: LA 403–425 For 5′ AATTTCCTCTCACTCATCCCAGACACAGGGTGGGGGCAGCGTCACAAAAAGCGCAAACTGGAGTCCACTGAG 3′, Rev 5′ GATCCTCAGTGGACTCCAGTTTGCGCTTTTTGGTGACGCTGCCCCCACCCTGTGTCTGGGATGAGTGAGAGGA 3′. Site directed mutagenesis was performed by QuikChange site directed mutagenesis (Stratagene) according to the manufactureŕs instructions and by using full length lamin A [36] as a template and primers carrying the corresponding point mutations to generate GFP lamin A R401C (1201C>T), G411D (1232G>A), G413C (1237G>T), V415I (1243G>A), R419C (1255C>T), L421P (1262T>C), R427G (1279C>G), Q432X (1294C>T).

### Cell culture and transfection

HaCaT, COS7 cells [37], [38], murine myoblasts (ATCC CRL-1772) and primary human fibroblasts obtained from Dr. M. Wehnert (Greifswald) [2] were grown in a humidified atmosphere at 37°C, 5% CO_2_ in high Glucose Dulbeccós modified Eagle's medium (DMEM) (SIGMA) supplemented with 2 mM glutamine, 2 mM penicillin/streptomycin and 10% FBS. Cells were transiently transfected by electroporation using Gene-Pulser®II (BioRad) at 180 V, 950 µF or by using the Amaxa cell line Nucleofector® kit (Lonza) according to the manufactureŕs instructions.

### His- and GST-tag pull down assays

GST or His tagged fusion proteins were expressed in *E. coli* XL1 blue. Bacteria were grown to an OD_600_ between 0.6 and 0.8 and protein expression over night at 20°C was induced by the addition of 0.5 mM Isopropyl-1-thio-D-galactopyranoside (IPTG). The bacteria were harvested, washed and lysed with STE buffer (10 mM Tris-HCl, pH 8.0, 50 mM NaCl, 1 mM EDTA) supplemented with protease inhibitors. Lysis was performed by mechanical shearing in a dounce homogenizer in the presence of 100 µg/ml lysozyme followed by 15 min incubation on ice. Lysates from bacteria expressing GST fusion proteins were additionally supplemented with Sarkosyl to a final concentration of 1.5%. All samples were sonicated and centrifuged at 16.000 rpm for 30 min. Supernatants were transferred into a new tube and lysates from bacteria expressing GST fusion proteins were supplemented with Triton X-100 to a final concentration of 2%. GST and His-tag fusion proteins were concentrated from bacterial lysates by adding glutathione Sepharose 4B or Ni-NTA beads on at 4°C. Beads were washed five times with PBS to remove unspecifically bound proteins. COS7 cells expressing the corresponding GFP plasmids were lysed with lysis buffer (50 mM Tris-HCl, pH 7.5, 150 mM NaCl, 1% Nonidet-P40, and 0.5% sodium deoxycholate) supplemented with protease inhibitors. For preclearing, lysates of COS7 cells expressing GFP fusion proteins were incubated with beads for one hour at 4°C followed by an on incubation with the corresponding GST- or Ni-NTA-beads bound fusion proteins. Finally beads were washed five times with PBS supplemented with protease inhibitors. SDS loading buffer was added and samples were heated for 5 min at 95°C and analysed by SDS-PAGE followed by Coomassie Blue staining or western blot.

### Immunofluorescence and microscopy

Cells were fixed on coverslips for 15 min in 4% paraformaldehyde in PBS followed by 5 min permeabilization in 0.5% Triton X-100 in PBS. Fixed samples were incubated for 30 min to 1 h in phosphate-buffered gelatine for blocking (PBS, 0.1% fish gelatine and 0.5% BSA) followed by incubation with primary antibodies or TRITC Phalloidin (Sigma) for 1 h at RT or overnight at 4°C followed by three washing steps with PBS, 5 min each. Appropriate secondary antibodies conjugated to Alexa 568 or Alexa 488 were applied. Nuclei were stained with 4,6-diamidino-2-phenylindole (DAPI). Samples were again extensively washed with PBS and fixed with gelvatol. Immunofluorescence analysis was performed as described [2].

### Antibodies

The following antibodies were used in this study; polyclonal rabbit anti Nesprin-2 pAbK1 [22], mouse monoclonal anti GFP K3-148-2, mouse mAb specific for Emerin (4G5, abcam), polyclonal rabbit anti lamin B1 (abcam), polyclonal rabbit anti lamin A (H-102, Santa Cruz).

### Quantification of western blot and Coomassie Blue stained gels

Quantification was performed by using the AlphaEaseFC software, version 4.0.0, Alpha Innotech Corporation. GFP lamin A signals were normalized against GST Nesprin-2 SR52,53 by generating the ratio of western blot GFP lamin A and Coomassie Blue GST Nesprin-2 signals. The ratio between WT GFP lamin A SR52,53 and GST Nesprin-2 was set to 100%. The percentage of enhanced or decreased binding between mutated GFP lamins and WT GST Nesprin-2 was set in correlation to the WT ratio.

### Heat shock experiments

Heat shock was performed by incubating cells plated on cover slips in 24 well plates in an incubator at 42°C for 15 min under otherwise normal cell culture conditions in a humidified atmosphere with 5% CO_2_. Afterwards cells were fixed by the addition of methanol and 10 min incubation at −20°C, followed by extensive washing and immunofluorescence analysis as described above.

### Protein structure prediction

aa sequences of human lamin A (P02545), *Musmusculus*lamin A (P48678) and lamin B (P20700) were taken from the UniProt database and aligned using the clustalW2 online program [39]. Aligned sequences were further processed by using ESPript 2.2 [40] for representation. For lamin A structural model construction, lamin A amino acids 351–490 that encompass a part of the N-terminal coil2B and the C-terminal globular domain were used in the MULTICOM server [41]. The templates used for modeling 1UFGA [42] (C-terminal immunoglobulin like domain of mouse Lamin A), 1IFRA (globular tail of human Lamin A), and 2LLA [43] (chain A of Mannose-6-phosphate/insulin-like growth factor II receptor) were taken from the PDB (Protein Data Bank). Detailed amino acid sequences and exchanges were generated by using SwissPDB Viewer v4.1.0 [44] and the molecular surfaces were generated by using pyMOL v1.3.

## Results

### C-termini of Nesprin-2 and lamin A interact

**Figure 1:**
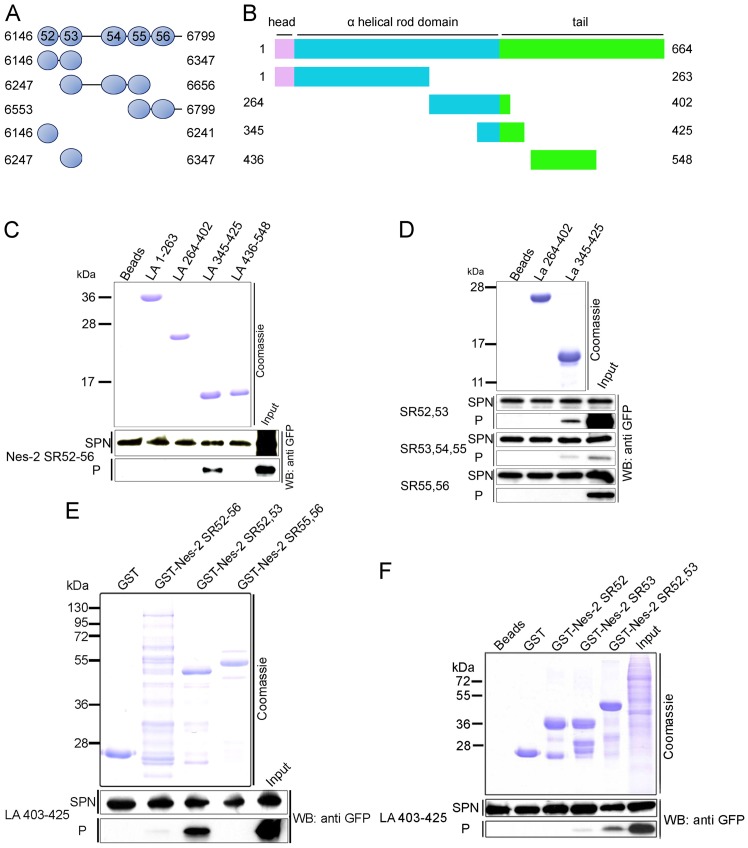
Nesprin-2 SR52,53 (aa 6146–6347) interacts with lamin A residues extending from position 403 to 425. **(A)** Schematic of GST Nesprin-2 fusion proteins used in this study. Aa positions refer to Nesprin-2 giant. (**B**) Schematic of His tag lamin A fusion proteins. (**C**) Bacterially expressed lamin A proteins bound to beads were incubated with lysates of COS7 cells expressing GFP Nesprin-2 SR52-56. The polypeptides used for pull down are shown in the upper panel. GFP-tagged Nesprin-2 SR52-56 was detected with mAb K3-184 (WB: anti GFP). The molecular weight markers are indicated on the left. GFP-tagged Nesprin-2 SR52-56 precipitates with lamin A polypeptides 345–425. No signals were detected for lamin A 264–402. For this reason we used these two lamin A proteins for our following experiments, one as a positive control and one as a negative control, to narrow the binding site of Nesprin-2 to lamin A. (**D**) Identification of the lamin A binding site in Nesprin-2. GFP Nesprin-2 polypeptides harboring individual SRs were expressed as GFP tagged proteins in COS7 cells (lower panels) and incubated with lamin A fusion proteins (upper panel, Coomassie Blue stained SDS PAGE, 15% acrylamide). (**E**) Interaction of GST Nesprin-2 fusion proteins bound to beads with GFP Lamin A aa 403–425. (**F**) Determination of the Nesprin-2 SR domain for interaction with lamin. Nesprin-2 SR52 and SR53 were expressed as GST fusion proteins and binding to GFP lamin A 403–425 was probed. For all experiments the use of equal protein amounts is demonstrated by Coomassie Blue stained SDS-PAGE. Equal amounts of GFP fusion proteins are shown by western blots of supernatants after coupling the GST and GFP fusion proteins. *Nes-2 SR* – Nesprin-2 Spectrin Repeats, *LA* – lamin A, *WB* – western blot, *SPN* – supernatant, *P* – pellet.

We defined here the exact binding sites between lamin A/C and Nesprin-2 by performing a series of *in vitro* pull down experiments with purified recombinant Nesprin-2 and lamin A proteins (Fig. 1A, B). GST tagged Nesprin-2 polypeptides we used for our studies were derived from the C-terminus encompassing aa 6146–6799 of human Nesprin-2 giant (Fig. 1A). We have described these polypeptides before as Nesprin-2 SRs 19–22 [22], however here we have renamed them according to a recent detailed structural analysis predicting almost the entire Nesprin-2 sequence is made from more or less well conserved spectrin repeats [24]. For lamin A we used polypeptides spanning amino acids 1–548 of human lamin A (Fig. 1B). Nesprin-2 SR 52–56 coprecipitated with lamin A amino acids 345–425 (Fig. 1C). Using lamin A polypeptides 264–402 and 345–425 which share an overlapping sequence (Fig. 1B) we found that Nesprin-2 SR 52–56 did not coprecipitate with lamin A 264–402, letting us conclude that the interaction site of lamin A for Nesprin-2 lies within the amino acids 403–425 of lamin A. To further define the interaction site lamin A fragments were incubated with GFP Nesprin-2 polypeptides expressed in COS7 cells. Nesprin-2 SR 52,53 showed the strongest interaction to lamin A, whereas no interaction was found for SR55, 56. A weak signal was detected for SR53, 54, 55 (Fig. 1D). To confirm the afore described interaction, we cloned the sequences containing the lamin A interaction site for Nesprin-2 (lamin A aa 403–425) into a GFP expression vector and incubated these proteins with purified Nesprin-2 polypeptides, which confirmed the interaction of lamin A to Nesprin-2 SR52,53 (Fig. 1E). Finally, we addressed the question if lamin A binds to SR52 or SR53 and found that Nesprin-2 SR53 is sufficient to precipitate GFP lamin A aa 403–425 from total cell lysates. The GFP lamin A 403–425 signal for SR53 was always much weaker compared to Nesprin-2 SR52,53 (Fig. 1F). Taken together aa 403–425 in lamin A and aa 6146–6347 corresponding to SR52,53 in Nesprin-2 mediate the interaction between both proteins.

### Laminopathy causing mutations reside in the Nesprin-2 binding site of lamin A

**Figure 2:**
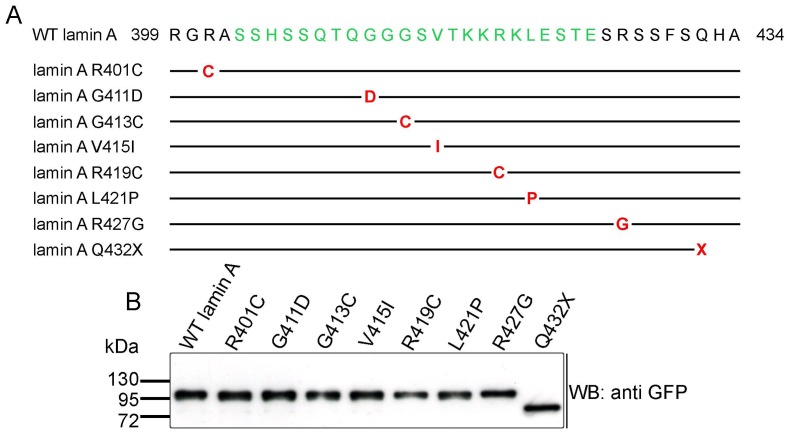
Schematic of lamin A mutations. (**A**) aa residues 399 to 434 of WT lamin A are shown. The interaction site of lamin A to Nesprin-2 is given in green. Below, the WT sequence mutations analysed in the present study are highlighted. Similarities to the WT sequence are shown as black lines, exchanged aa are indicated in red. The mutation Q432X leads to a stop codon (X). (**B**) Western blot analysis of GFP lamin A fusion proteins. The proteins were transiently expressed in COS7 cells, lysates were analysed by SDS-PAGE followed by western blot with mAb K3-184 that confirmed the predicted molecular weights (given in kDa on the left). *WB* – western blot.

Next we addressed the question if relevant mutations have been described within or nearby the Nesprin-2 binding site in lamin A. We found eight mutations in *LMNA* [R401C (1201C>T), G411D (1232G>A), G413C (1237G>T), V415I (1243G>A), R419C (1255C>T), L421P (1262T>C), R427G (1279C>G), Q432X (1294C>T), (Fig. 2A), for references see introduction]. All mutations reside within a region of lamin A that is encoded by *LMNA* exon 7 and all are point mutations causing single nucleotide exchanges. Seven mutations result in amino acid changes whereas mutation Q432X (1294C>T) results in a stop codon (Fig. 2A). The predicted molecular weight for GFP WT lamin A and all mutations is ∼100 kDa, for the truncated GFP lamin A Q432X protein the predicted molecular weight is ∼76 kDa. In western blots all lamin A variants are detectable at the predicted molecular weights (Fig. 2B).

### Influence of *LMNA* mutations on the distribution of interaction partners

**Figure 3:**
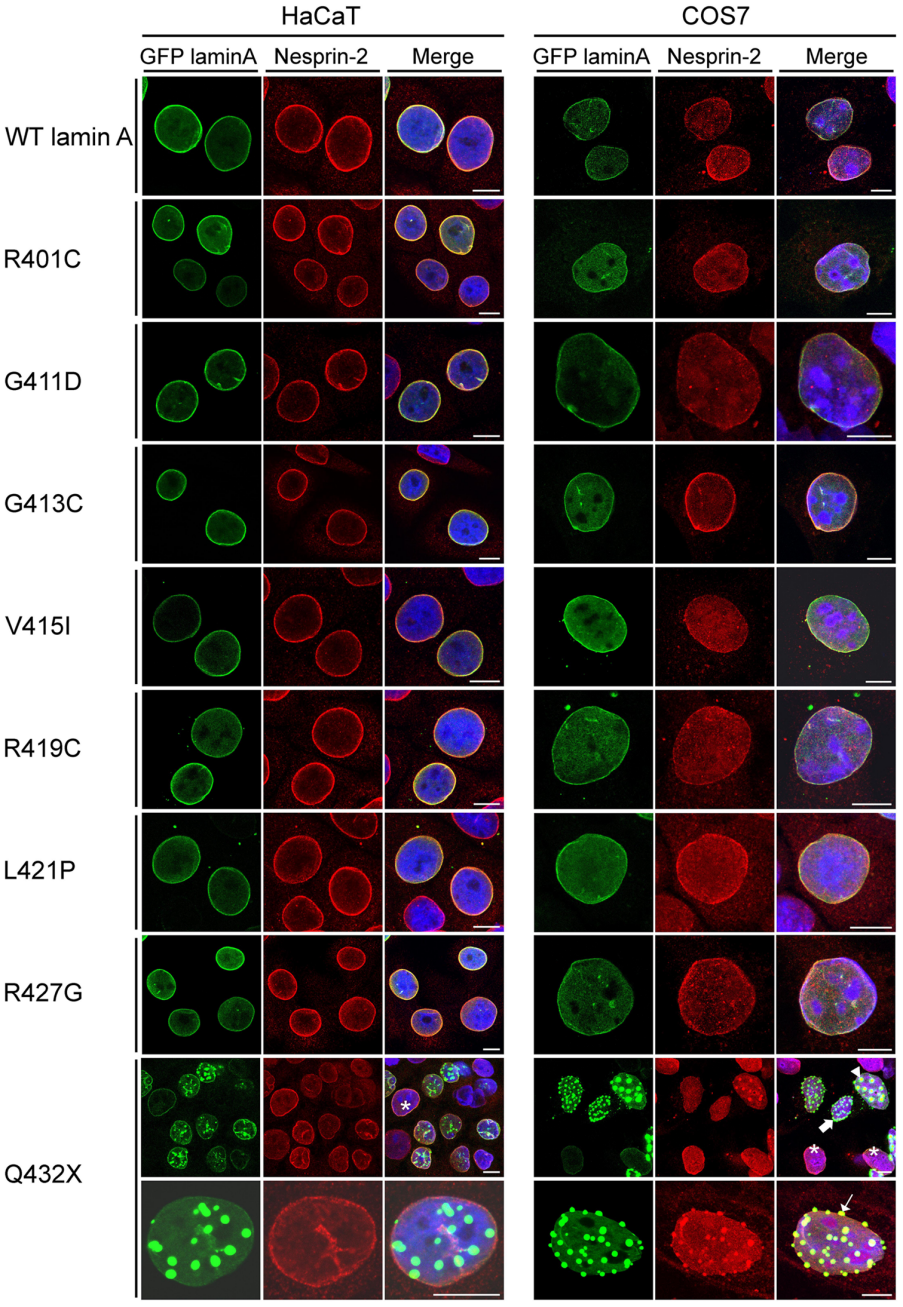
Most mutations in lamin A do not alter the distribution of Nesprin-2. The distribution of Nesprin-2 in transiently GFP-lamin A wild type (WT) and mutant protein expressing COS7 and HaCaT cells was analysed by immunofluorescence. For GFP lamin A Q432X two panels are shown indicating different distribution and presence of smaller and larger GFP lamin A Q432X aggregates. Upper panel, asterisks points out cells in which lamin A Q432X proteins localize indistinguishable from the WT proteins. Lamin A Q432X proteins form aggregates with varying extend. In COS7 cells endogenous Nesprin-2 is sequestered into strong aggregates (arrowhead) whereas smaller aggregates show no obvious Nesprin-2 accumulation (bold arrow). In HaCaT cells Nesprin-2 is largely absent from the aggregates (HaCaT, Q432X, lower panel). Aggregates are present along the NE (COS7, lower panel, thin arrow). Merged pictures contain overlays of single stainings and DAPI. Scale bar, 10 µm.

To test if mutations in lamin A cause changes in NE protein assemblies, the distribution of WT and mutated GFP lamin A and endogenous LINC complex components was studied in immunofluorescence analysis. We utilized HaCaT and COS7 cells which differ with respect to their morphology and the expression levels of Nesprins. Previous work from our group demonstrated that HaCaT keratinocytes express higher Nesprin-2 protein levels compared to fibroblast cells [11], [45]. The fibroblasts like COS7 cells express lower levels of Nesprin-2 as compared to HaCaT keratinocytes. We found all GFP fusion proteins localizing along the nuclear envelope like WT lamin A. GFP lamin A Q423X proteins showed a different distribution. In COS7 as well as in HaCaT cells Q432X localized along the NE (Fig. 3, asterisk), additionally, GFP lamin A Q432X proteins formed aggregates that seemed to appear along the NE rather than inside the nucleus (Fig. 3, thin arrow). In HaCaT cells Nesprin-2 was largely unaffected by these aggregates and showed the typical rim staining (Fig. 3). Only when large aggregates were present Nesprin-2 was also recruited to these aggregates (data not shown). By contrast, in COS7 cells Nesprin-2 was sequestered into large lamin A aggregates (Fig. 3, arrowhead). In cells with intermediate sized aggregates one can observe both, a recruitment (Fig. 3, thin arrow) and an absence of Nesprin-2 (Fig. 3, bold arrow) in the aggregates. Different distributions were observed for further NE components like lamin B1 (Fig. S1) or Emerin (Fig. S2). In HaCaT cells lamin B1 was present only in large aggregates, whereas in COS7 it appeared in both small and large aggregates. Emerin was present in large aggregates formed in both cell lines to comparable extend. Endogenous lamin A was also sequestered into GFP lamin A Q432X aggregates (Fig. S3).

### Mutations in *LMNA* modulate binding affinities of lamin A for Nesprin-2

**Figure 4:**
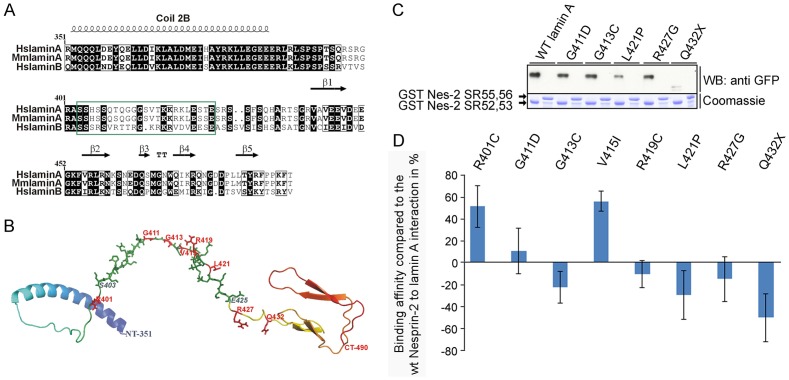
The interaction site of lamin A to Nesprin-2 is in a loop and mutations in *LMNA* modulate the interaction to Nesprin-2. (**A**) Amino acid alignment of a human lamin A/C amino acids 351–490 (HsLaminA; uniprot accession number P02545) and the corresponding sequence in human lamin B (HsLamin; P20700) and *Mus musculus* lamin A (MmLaminA; P48678). Secondary structure elements are shown on top of the alignment and the lamin A region that interacts with Nesprin-2 is boxed in green. Conserved residues are highlighted black, similar residues are boxed. (**B**) Prediction of the three dimensional structure of human lamin A aa 351–490. The three dimensional structure of human Lamin A^351–490^ was predicted by using multiple PDB structures as templates (1UFGA, 1IFRA, and 2LLA) in the MULTICOM server [41]. The lamin A interaction site aa 403–425 to Nesprin-2 is highlighted in green and aa 403 and 425 are pointed out in blue. Amino acids that targets for the mutations analysed here are highlighted in red. The structure prediction was generated by using pyMOL v1.3. (**C**) The binding properties between WT GST Nesprin-2 SR52,53 and GFP lamin A mutations were analysed by pull down experiments. COS7 cells expressing WT or mutated GFP lamin A proteins were lysed and incubated with recombinant GST Nesprin-2 SR52,53 proteins. GST Nesprin-2 SR55,56 proteins were used as negative controls. (**D**) WT and mutant GFP lamin A proteins show distinct binding properties to WT GST Nesprin-2 SR52,53. The zero baseline represents the 100% binding affinity between WT GST Nesprin-2 SR52,53 and GFP lamin A (for details see materials and methods). Deviations caused by distinct mutations in *LMNA* are given in percent. Each mutation was analysed by four to seven independent experiments.

Next we addressed the question if the lamin A mutations analysed here modulate the affinity to GST Nesprin-2 protein complexes. For this, we first made structural predictions of the region in lamin A that interacts with Nesprin-2 and found that the interaction site in lamin A is located in a loop (Fig. 4B). This loop connects the α helical rod domain and the globular domain at the C-terminus (Fig. 4A). The affected amino acids should therefore be accessible for interactions. The impact of the amino acid exchanges caused by the lamin A mutations analysed here reach from charge neutrality to the generation of positively or negatively charged residues (Fig. S4). Recombinantly expressed GST Nesprin-2 SR 52,53 was used as positive and GST Nesprin-2 SR55,56 as negative control. The proteins were incubated with lysates of COS7 cells expressing the corresponding GFP lamin A proteins (Fig. 4C). Mutated lamin A proteins were precipitated in varying amounts with GST Nesprin-2 SR52,53 (Fig. 4C, shown for lamin A mutations G411D, G413C, L421P, R427G and Q432X). The lamin A mutations R401C and V415I were precipitated at higher amounts compared to WT GFP lamin A, whereas only small amounts of Q432X were precipitated by GST Nesprin-2 SR52,53 (Fig. 4D). All further mutations were precipitated at levels comparable to WT GFP lamin A.

### Mutations in lamin A cause the formation of misshapen nuclei after heat exposure

**Figure 5:**
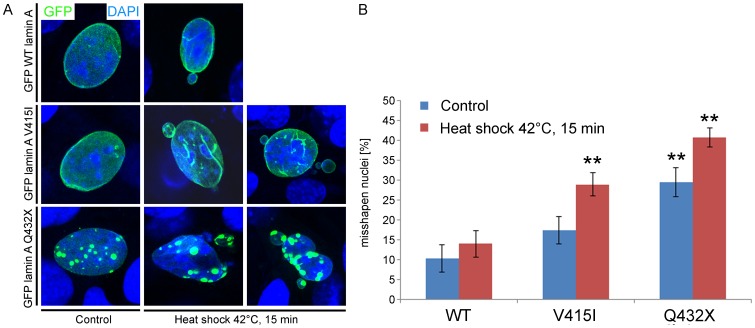
Lamin A mutations V415I and Q432X trigger nuclear deformations in heat shock experiments. C2C12 murine myoblast cells transiently expressing GFP WT, V415I or Q432X lamin A were exposed to a 15 minute heat shock at 42°C. After fixation the nuclear morphology was analysed by immunofluorescence (**A**) and statistically evaluated (**B**). Cells expressing GFP lamin A WT were used for reference. 600 nuclei each were analysed. Cells expressing GFP lamin A Q432X showed significant nuclear deformations already before the heat shock. P-Values of less than 0,001 are defined as highly significant (**).

Laminopathies often affect tissues that are under pronounced mechanical strain like skeletal muscle or the heart. A hallmark of laminopathies is the presence of dysmorphic nuclei, impaired mechanical properties and stiffness of the nucleus [46]. We exemplarily explored the effect of two lamin A mutations, lamin A V415I and lamin A Q432X on nuclear stability in heat stress experiments since laminopathic cells have been shown to be more prone to heat induced nuclear deformations [47]–[49]. The mutations were chosen because they exhibited significantly different binding affinities and showed the most aberrant distribution (Fig. 3, 4). C2C12 murine myoblasts were exposed to a 15 minutes heat shock and the percentage of misshapen nuclei was assessed before and after this treatment. No significant changes were observed among WT lamin A expressing cells before (10,33%±4,08 misshapen nuclei) or after (14,0%±4,05) heat shock. Stronger increases in the number of misshapen nuclei compared to the WT were observed for the lamin A mutations V415I (17,5%±4,08 before and 29,0%±3,41 after heat shock) and Q432X (29,5%±4,37 before and 40,83%±2,86 after heat shock). The extent of nuclear deformations caused by the lamin A mutations V415I and Q432X was similar in heat shock experiments performed in primary human fibroblasts (Fig. S5). Both lamin A mutations showed nuclear deformations characteristic for laminopathies including an uneven nuclear shape and nuclear blebbing (Fig. 5A). Nuclear deformations were even more severe in cells expressing the mutant lamin A Q432X compared to V415I (Fig. 5A, B).

### The perinculear actin cap remains unaffected by the lamin A mutations Q432X and V415I

**Figure 6:**
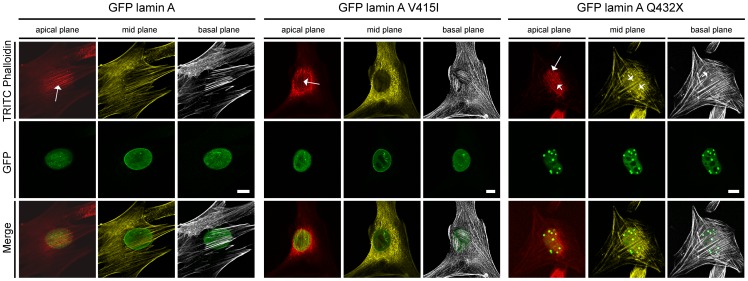
The perinuclear actin cap is not altered in the presence of GFP lamin A Q432X or V415I. C2C12 cells transiently expressing WT GFP lamin A, the mutant proteins Q432X or V415I were stained with DAPI and TRITC phalloidin. The stainings were documented by using a confocal microscope and pictures were taken at the apical plane (red), the mid plane (yelow) and the basal plane (gray). Actin cap fibers are present in control or mutant lamin A expressing cells (long arrow). TRITC phalloidin was present in some GFP lamin A Q432X aggregates (short arrow).

Since the lamin A mutation Q432X shows the most severe impact on interaction partners or nuclear morphology, we additionally addressed the question if the perinuclear actin cap is altered in the presence of mutated protein. The perinuclear actin cap has functions in mechanosensing and maintaining interphase nuclear shape [50], [51]. Additionally the lamin A mutations V415I was chosen because it strongly precipitates with Nesprin-2 polypeptides. In both, lamin A Q432X or V415I expressing cells, filaments forming the actin cap aligned regularly along the interphase nucleus without obvious alterations compared to GFP lamin A WT expressing cells (Fig. 6, long arrow). In some of the stronger GFP lamin A Q432X aggregates we could observe TRITC phalloidin positive Q432X aggregates (Fig. 6, short arrow). For the other mutations no obvious alterations in the perinuclear actin organizations were observed in 3T3 cells (data not shown).

### The lamin A Q432X mutation causes alterations in the chromatin and SRBP1 misplacement

**Figure 7:**
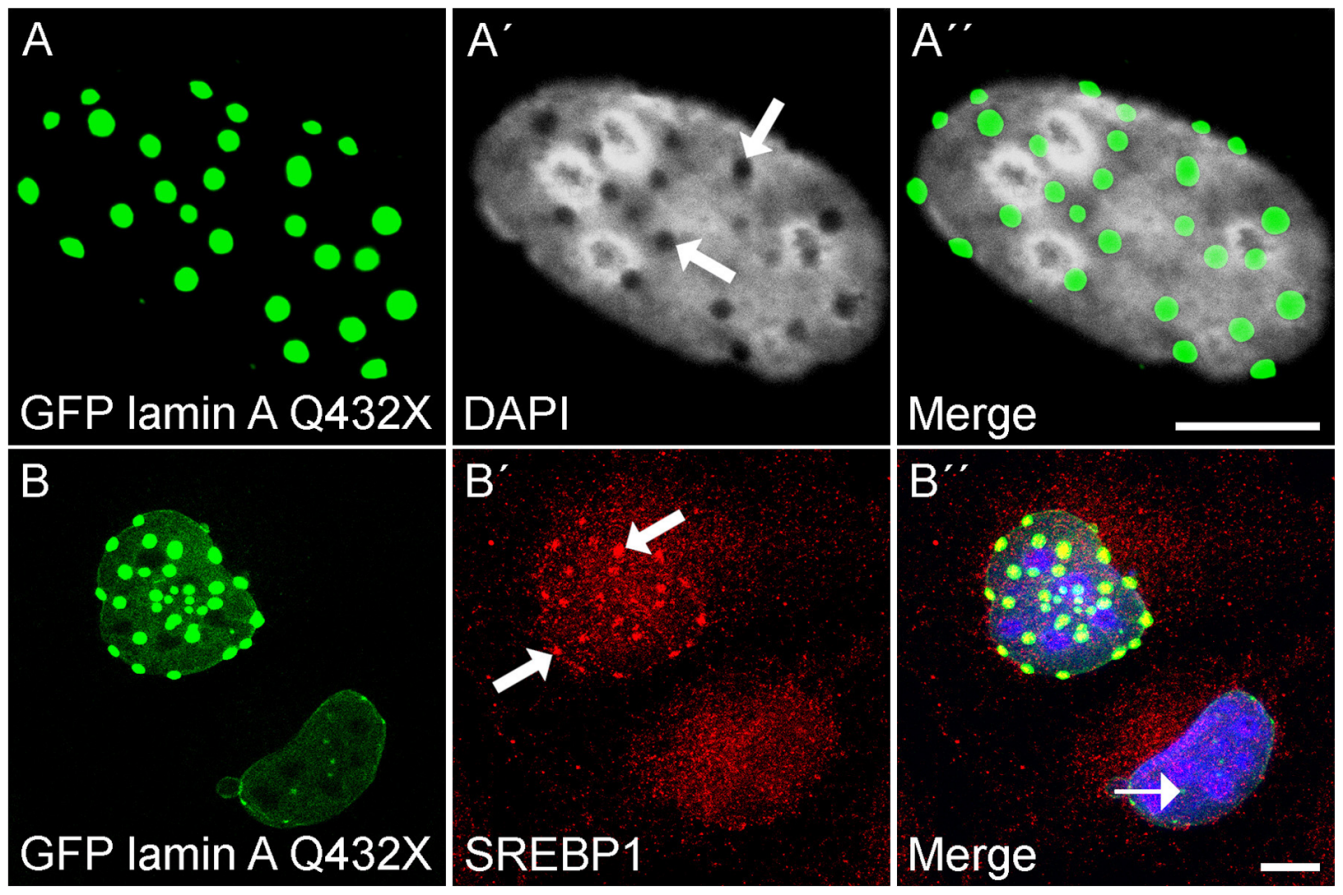
GFP lamin A Q432X aggregates displace chromatin and sequester SREBP1. COS7 cells transiently expressing GFP lamin A Q432X were stained with DAPI (**A–A**) and SREBP1 and DAPI (**B–B**). (**A**) At sides of strong aggregates the DAPI staining showed gaps (A, arrow). (B) Endogenous SRBP1 was sequestered into strong aggregates (B, arrow), rather than into smaller aggregates (B, thin arrow). Scale Bar: 10 µm.

The lamin A mutation Q432X showed the most variable distribution along the NE that included the formation of aggregates and the sequestration of LINC complex components combined with decreased binding affinity to Nesprin-2 polypeptides (Fig. 3, 4). We further explored the lamin A mutation Q432X to explain the role of this particular mutation in the formation of idiopathic dilated cardiomyopathy. NE proteins control gene expression by modulating the organization of chromatin or regulating the accessibility of transcription factors to their nuclear targets [52]. To explore the impact of the aggregates formed by GFP lamin A Q432X on chromatin structures, cells transiently expressing the mutated protein were stained with the DNA dye DAPI. At sites of strong aggregates the DAPI staining showed gaps indicating alterations in the chromatin structure (Fig. 7A ´, arrow). Such a staining pattern was not seen in control cells. The opposite effect was detected for SREBP1 (sterol regulatory element-binding protein 1, also SRBP1 or SREBF1 (sterol regulatory element-binding transcription factor 1)), a transcriptional activator that belongs to the basic-helix-loop-helix-leucine zipper family of transcription factors. Its functions were originally described in cholesterol and fatty acid metabolism [53], however recent findings additionally demonstrated its expression [54] and function in muscle where SRBP1 transcription factors regulate the expression of hundreds of genes [55] that affect muscle size and mass. SREBP1 is primarily sequestered into large aggregates of Q432X mutated lamin A proteins (Fig. 7B ´, arrow) and less so into smaller aggregates (Fig. 7B ´´, thin arrow). Taken together our data point towards an impact of the mutation Q432X on the topology of chromatin and transcription factor distribution.

## Discussion

Laminopathies are rare heterogenic human diseases with complex genotype phenotype relationships that can be classified into muscle diseases that preferentially affect skeletal or cardiac muscles, neuropathies that involve motor and sensory neurons, lipodystrophies that cause alterations in adipose tissues and premature ageing diseases [56]. In earlier studies we had identified the Nesprin-2 C-terminal amino acid sequence 6146–6799 corresponding to Nesprin-2 SRs 52–56 as binding site for lamin A, here we mapped it between aa 6146–6347 (SR52,53) and the binding sites of Nesprin-2 to lamin A to aa 403–425. The previously reported interaction site between lamin A aa 243–387 [22] could not be confirmed in the present study which might be a consequence of different experimental procedures or sterical properties of the lamin A peptides that were used. Aim of this study was to analyze the impact of laminopathy causing mutations on the interaction properties between lamin A and Nesprin-2.

Laminopathies often occur in tissues that are under significant mechanical stress as it occurs in skeletal or cardiac muscles. Therefore it is of particular importance to analyze the interaction among core NE proteins and their NE distributions. We describe here eight *LMNA* mutations. Interestingly they can be classified as lipodystrophy (G411D, R419C, L421P) and skeletal and cardiac muscular dystrophies causing mutations (R401C, G413C, V415I, R427G, Q432X). Mutations triggering premature ageing diseases or neuropathic phenotypes have not been described in this binding region to Nesprin-2 so far, but cannot be excluded based on the progress in the field of DNA sequencing. All GFP lamin A proteins that were analysed here localize along the NE. In agreement with earlier studies of ectopically overexpressed WT and mutant lamin A proteins, we observed some cells that showed altered localizations. The degree of altered localization patterns differed between HaCaT keratinocytes and COS7 fibroblasts. HaCaT cells seem to have a more stable nuclear morphology and are less prone to nuclear deformation than fibroblasts which confirms earlier findings and might be attributed to lower Nesprin expression in fibroblast cells [11]. We excluded phenotypes that might be related to common morphological variations in these cells.

GFP lamin A Q432X was the only mutation that caused significant alterations in the localization pattern along the nuclear envelope and formed strong aggregates. The GFP lamin A Q432X aggregates had a similar morphology as the ones caused by mutations in *LMNA* that have been mapped at different positions along the lamin A protein. Aggregate formations have been described e.g. for the lamin A mutations D192G, N195K, M371K, R386K, R482L [57], [58]. Bechert and colleagues analysed the distribution of WT, G465D, R482L and R527P lamin A and found that 24 hours after transfection the proteins localized to the nucleus or the NE. A prolonged expression resulted in the formation of aggregates with varying intensities among mutated or WT lamin A [59]. The situation was different for GFP lamin A Q432X. 24 hours after transfection the formation of aggregates in the majority of cells was observed in sharp contrast to all other mutant or WT lamin A proteins analysed here. As no primary cells were available we could not address the question if lamin A Q432X proteins in patient cells form aggregates. We analysed the distribution of further NE proteins along these aggregates and found that Nesprin-2, Emerin, Lamin B1, endogenous lamin A were sequestered into the aggregates. Considering the reduced binding ability between GFP Nesprin-2 SR 52–56 and lamin A Q432X, at first sight it appears surprising to see Nesprin-2 accumulations in these aggregates. A reasonable explanation for this is the enhanced presence of NE proteins Emerin or endogenous lamin A that might recruit endogenous Nesprin-2 into the aggregates.

Even though we did not observe differences in the subcellular localization of most lamin A mutations analysed here, we further explored the effect of distinct mutations in *LMNA* on the binding abilities between Nesprin-2 and lamin A by pull down experiments. Of note, such experiments do not examine the direct interaction between Nesprin-2 and lamin A, rather they show how mutated GFP lamin A proteins precipitate with GST Nesprin-2 polypeptides from whole cell lysates. Interestingly the binding site of lamin A to Nesprin-2 resides within a loop that harbors multiple potential phosphorylation sites [60] indicating that protein interactions in this part of lamin A occur highly regulated. The advantage of the experimental setting we have chosen is that GFP lamin A - GST Nesprin-2 SR52,53 protein complexes are precipitated from otherwise normal whole cell lysates which allows studying binding characteristics in the presence of additional LINC complex components expressed in these cells. Interestingly we observed a wide range of variations in the amounts of GFP lamin A proteins that were precipitated in pull down experiments that reached from enhanced (e.g. R401C, V415I) to decreased signals (e.g. Q432X) compared to WT GFP lamin A. Further mutations analysed here showed mild alterations. The truncation in Q432X does not include the amino acids 403–425 that mediate the lamin A interaction to Nesprin-2. However lamin A Q432X precipitates in lower amounts with GST tagged Nesprin-2 polypeptides. A reasonable explanation for this is that the loss of the C-terminus of lamin A causes alterations in the three dimensional protein structure which might make the interacting amino acids less accessible. Over all these findings contribute to the explanation how distinct mutations in ubiquitously expressed proteins like lamin A lead to the formation of diseases with strong variations in their clinical manifestations as it is known for laminopathies. Distinct mutations in *LMNA* have distinct effects on the formation of NE protein assemblies as it is shown here for interaction between the core NE components Nesprin-2 and lamin A. However the impact of distinct mutations on the interaction between Nesprin-2 and lamin A is not reflected in the aspect of nuclear stability. On the protein level lamin A V415I causes an enhanced interaction with Nesprin-2, whereas the lamin A Q432X interaction is weaker compared to the WT. At the immunofluorescence level they show differential distributions as well. Lamin A V415I is indistinguishable from the control, lamin A Q432X causes the formation of aggregates (Fig. 3). However, both mutations cause cardiomyopathic phenotypes, lone atrial fibrillation is caused by lamin A V415I and idiopathic dilated cardiomyopathy by lamin A Q432X. When we expressed both mutations in murine myoblasts or primary fibroblasts and treated them with a heat shock at 42°C both mutations led to a significant increase in the numbers of misshapen nuclei which shows that each mutation has additional distinct effects on NE protein assemblies. The underlying molecular mechanisms for the discrepancy between the contradictory behavior of the mutations V415I and Q432X in pull down experiments and a similar tendency in the heat stress experiments might be related to the complexity of NE protein assemblies. Lamins exhibit tripartite organization and are composed of an N-terminal head, a central rod and a C-terminal tail. The tail region that is missing in Q432X might impair the assembly of lamins into a meshwork and thus lead to pronounced nuclear fragility. On the other side the mutation V415I precipitates stronger with GST Nesprin-2 polypeptides indicating that this mutation modulates the binding properties among the NE protein network that finally results in enhanced nuclear fragility.

Nesprin-2 is an actin binding protein that connects the NE to the perinuclear cytoskeleton. Recently the Wirtz group identified a perinuclear actin cap that consists of highly organized actin fibers closely connected to the apical surface of the interphase nucleus [51]. LINC complexes physically connect the actin cap to the NE, and the actin cap is required to maintain the interphase nuclear shape and is likely involved in mechanosensing and mechanotransduction. Malfunctions in the actin cap might explain the pathological role of distinct lamin A mutations. An intact lamina and LINC complexes are necessary for a functional actin cap [50]. In GFP lamin A Q432X and V415I expressing cells the overall actin cap structure remained unaffected (Fig. 6). In lamin A Q432X expressing cells we observed a subset of phalloidin positive lamin A Q432X aggregates. The fact that not all aggregates were phalloidin positive might be explained with the highly dynamic nature of the actin cap fibers that might form only in response to mechanical strain [51]. It remains to be determined if the observed changes in the actin structure lead to disturbed mechanotransduction from the extracellular milieu to the nucleus and thus contribute to the disease phenotype.

Alterations in the binding affinities between Nesprin-2 and lamin A have consequences on the NE protein network that are transmitted to further binding partners. Since lamins localize along the inner surface of the INM and Nesprins as type II transmembrane proteins can be integrated into the INM or the ONM, the interaction between both proteins occurs along the INM and the functional impact of altered affinities reaches into the nucleus. Interestingly most lamin interactions have been mapped to the C-terminus [8] and therefore overlap with the interaction side for Nesprin-2. Alterations in the binding affinities between both proteins therefore make lamins more or less accessible for other interactions. NE proteins have been described in maintaining chromosomal organization to provide a chromatin environment that is favorable or unfavorable for gene expression in which the position of a gene is related to its expression level [52], [61]. Lamins directly interact with DNA or Histones. The DNA binding site of lamins A/C was mapped to aa 411–553 [62] and aa 396–430 interact with histones [20]. Both sites are close to or part of aa 403–425 that interact with Nesprin-2 and variations in the lamin adhesion to Nesprin-2 could thus affect chromosomal arrangements and consequentially gene expression. Mutated Q432X lamin A proteins show the most severe impact on chromosome structures by the formation of gaps that are formed in the otherwise even DNA staining at sides of aggregates.

NE proteins additionally have functions in controlling the temporal and spatial accessibility of transcription factors to their nuclear targets. Lamins for example play a role in FOS or SREBP1 [18], [19] signaling and Nesprins are part of SMAD, FOS and Wnt signal transduction [13], [14]. Mutated lamin A Q432X proteins form aggregates and sequester LINC complex components. To further explore the pathological role of this mutation, we analysed the impact of the mutated proteins on the subcellular distribution of the transcription factor SREBP1 that is a known interaction partner of lamin A. The interaction site of lamin A to SREBP1 lies between amino acids 389–664 and therefore overlaps with the binding lamin A site to Nesprin-2 [19]. SREBP1 belongs to a group of ubiquitously expressed transcription factors with a wide spectrum of functions reaching from cholesterol and fatty acid metabolism to the regulation of muscle size and protein content [53], [63]. Furthermore SREBP1 transcription factor regulate the expression of proteins involved in controlling muscle contractility like Titin or Troponins [63], [64]. SREBP1 exists as an inactive precursor attached to the ER membrane and the NE. Cleavage of the SREBP1 precursor is initiated by sterol deficiency that releases the N-terminal part as a mature protein from the membrane that is translocated into the nucleus where it binds to the SRE1 (sterol regulatory element-1) DNA sequence in the promotor region of target genes [65], [66]. Malfunctions in SREBP1 target genes lead to the formation of dilated cardiomyopathies [67], [68]. It remains to be determined to which degree nuclear instability or aggregates formed by lamin A Q432X proteins contribute to the pathological role of this particular mutation in the formation of idiopathic dilated cardiomyopathy.

## Supporting Information

Figure S1 **Most mutations in lamin A do not affect the distribution of Lamin B1.** The distribution of endogenous lamin B1 was analysed in HaCaT and Cos7 cells transiently expressing GFP lamin A WT or mutated proteins. All mutated GFP lamin A proteins are present at the nuclear envelope like WT lamin. An exception is the truncation mutation Q432X that additionally forms aggregates of varying size. In HaCaT cells endogenous lamin B1 appears in strong aggregates. In COS7 cells the endogenous lamin B1 protein is sequestered into smaller aggregates. The merge contains the overlay of the single stainings and DAPI. Scale bar, 10 µm.
(TIF)

Figure S2 **Most mutations in lamin A do not affect the distribution of Emerin.** Distribution patterns of endogenous Emerin were analysed in HaCaT and COS7 cells transiently expressing WT or mutated GFP lamin A proteins. Merged pictures contain overlays of the single stainings and DAPI. Scale bar, 10 µm.
(TIF)

Figure S3 **Endogenous lamin A colocalizes with GFP lamin A Q432X.** HaCaT cells transiently expressing GFP lamin A Q432X were stained for lamin A with antibody. The epitope of this antibody is located in the C-terminus of lamin A that is missing in lamin A Q432X. The merge consists of the green, red signal and DAPI. Scale bar, 10 µm.
(TIF)

Figure S4 **Molecular surface properties of WT lamin A and laminopathy causing lamin A mutantions.** The figure shows a comparison of the surface rendering of WT lamin A aa 403–425 (left) and the same sequence including all lamin A mutations analysed here (right). Highly positive and negatively charged residues are shown in blue and red, respectively. Black arrowheads point on positively charged groups that are lost due to mutations. The red arrowhead points on a negatively charged group that is inserted by the lamin A mutation G411D.
(TIF)

Figure S5 **Lamin A mutations V415I and Q432X cause nuclear deformations in heat shock experiments.** Human fibroblasts transiently expressing GFP WT, V415I or Q432X lamin A were exposed to a 15 minute heat shock at 42°C and fixed immediately to evaluate nuclear morphology by immunofluorescence (**A**) followed by and statistic analysis (**B**). Cells transiently expressing GFP lamin A WT were used as a reference. Two independent experiments were performed and 300 nuclei each were analysed. Nuclei from cells expressing GFP lamin A Q432X showed significantly higher amounts of deformations already before heat shock. P-Values of less than 0,01 are defined as significant (*) and below 0,001 as highly significant (**).
(TIF)
