# Supplementary figures and images for: Mutations in LMNA Modulate the Lamin A - Nesprin-2 Interaction and Cause LINC Complex Alterations
Source: PLoS One. 2013 Aug 20;8(8):e71850. doi: 10.1371/journal.pone.0071850 (PMC3748058; doi:10.1371/journal.pone.0071850)

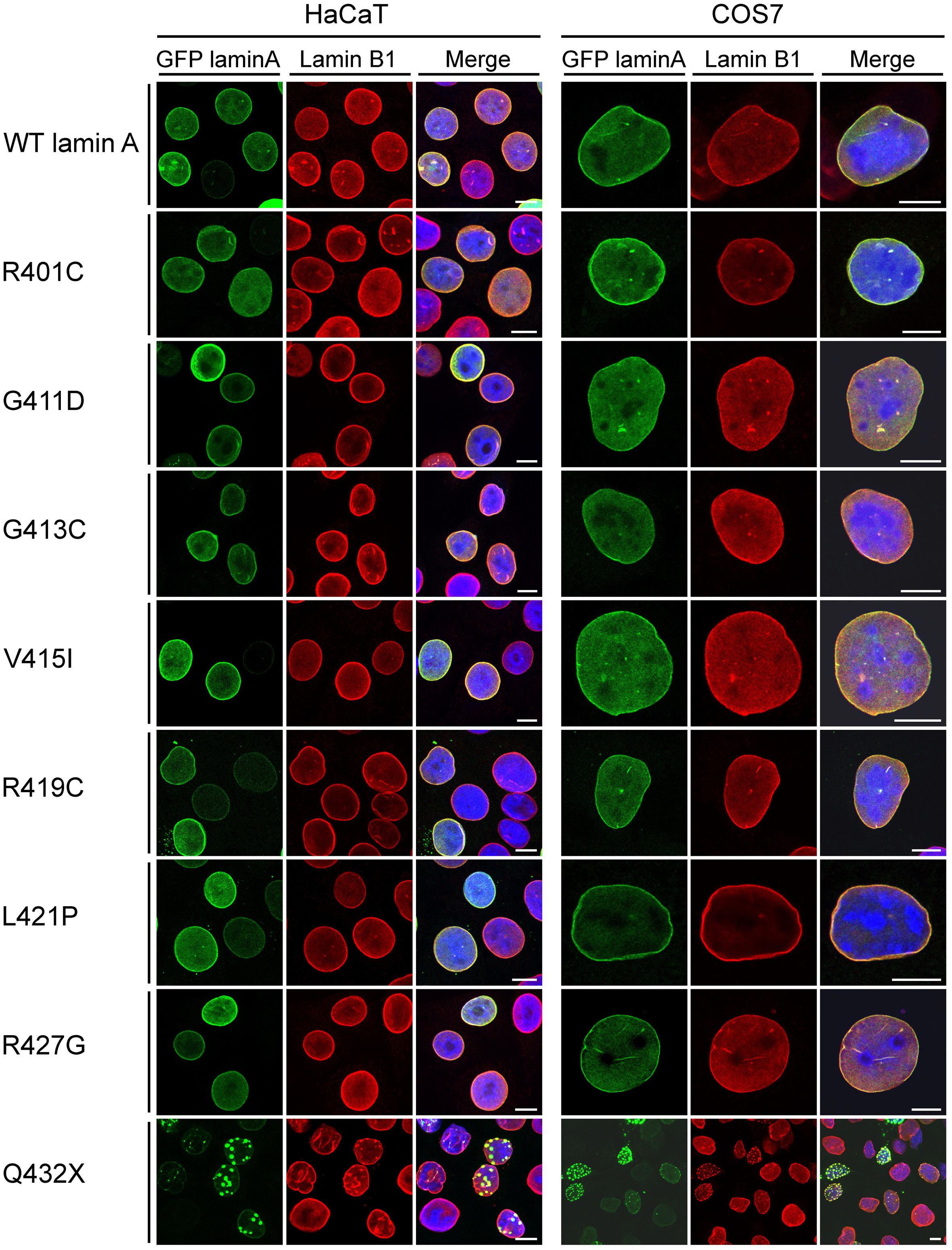

Supplement: Figure S1 — Most mutations in lamin A do not affect the distribution of Lamin B1. The distribution of endogenous lamin B1 was analysed in HaCaT and Cos7 cells transiently expressing GFP lamin A WT or mutated proteins. All mutated GFP lamin A proteins are present at the nuclear envelope like WT lamin. An exception is the truncation mutation Q432X that additionally forms aggregates of varying size. In HaCaT cells endogenous lamin B1 appears in strong aggregates. In COS7 cells the endogenous lamin B1 protein is sequestered into smaller aggregates. The merge contains the overlay of the single stainings and DAPI. Scale bar, 10 µm. (TIF) [file pone.0071850.s001.tif]

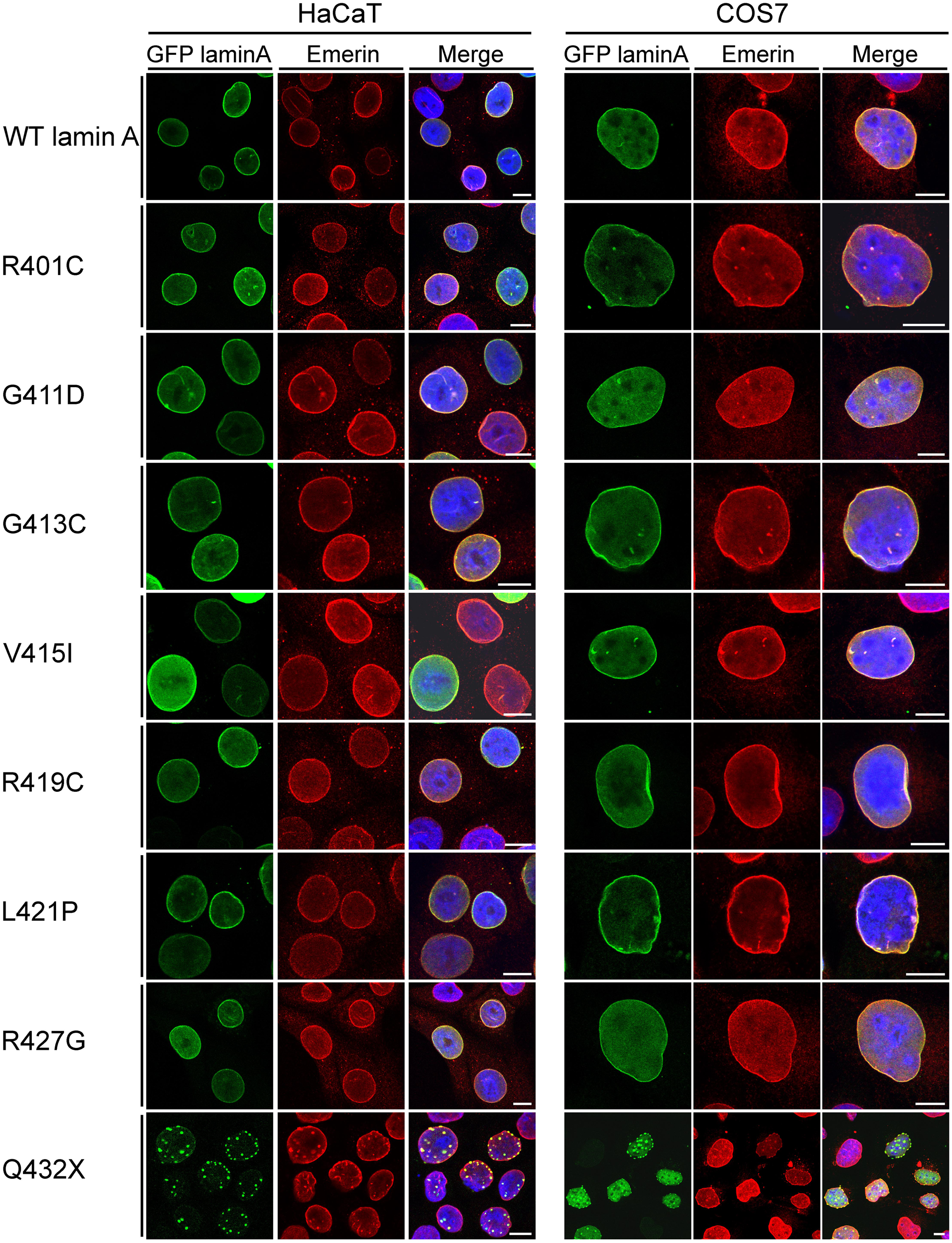

Supplement: Figure S2 — Most mutations in lamin A do not affect the distribution of Emerin. Distribution patterns of endogenous Emerin were analysed in HaCaT and COS7 cells transiently expressing WT or mutated GFP lamin A proteins. Merged pictures contain overlays of the single stainings and DAPI. Scale bar, 10 µm. (TIF) [file pone.0071850.s002.tif]

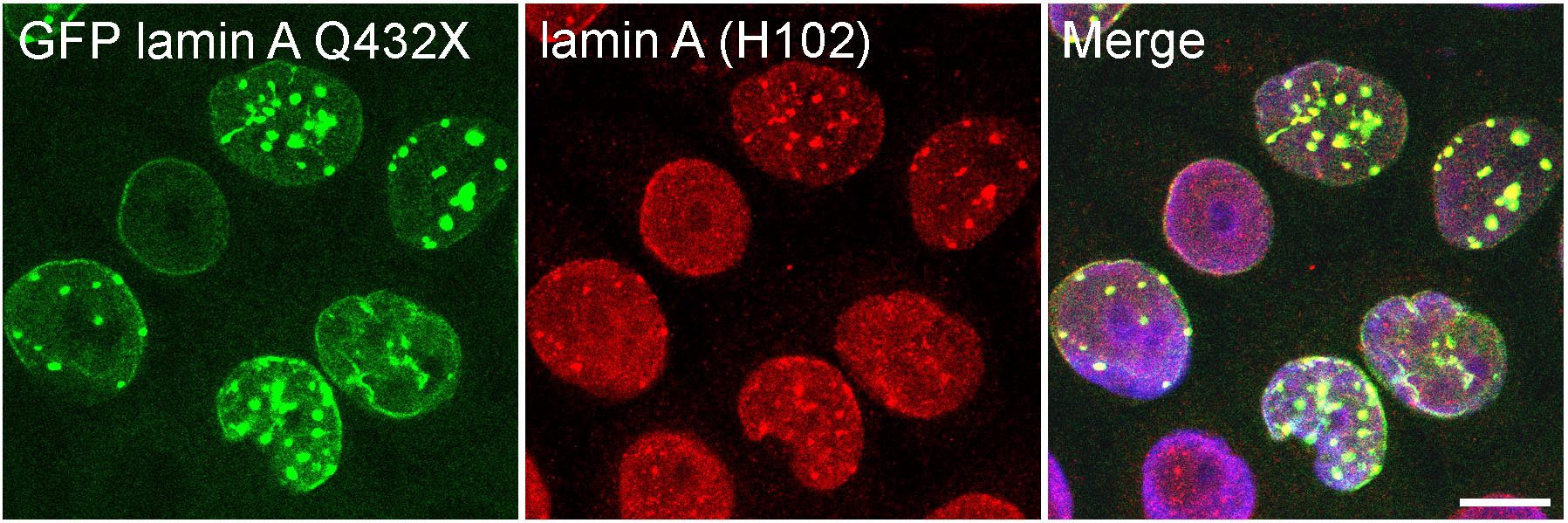

Supplement: Figure S3 — Endogenous lamin A colocalizes with GFP lamin A Q432X. HaCaT cells transiently expressing GFP lamin A Q432X were stained for lamin A with antibody. The epitope of this antibody is located in the C-terminus of lamin A that is missing in lamin A Q432X. The merge consists of the green, red signal and DAPI. Scale bar, 10 µm. (TIF) [file pone.0071850.s003.tif]

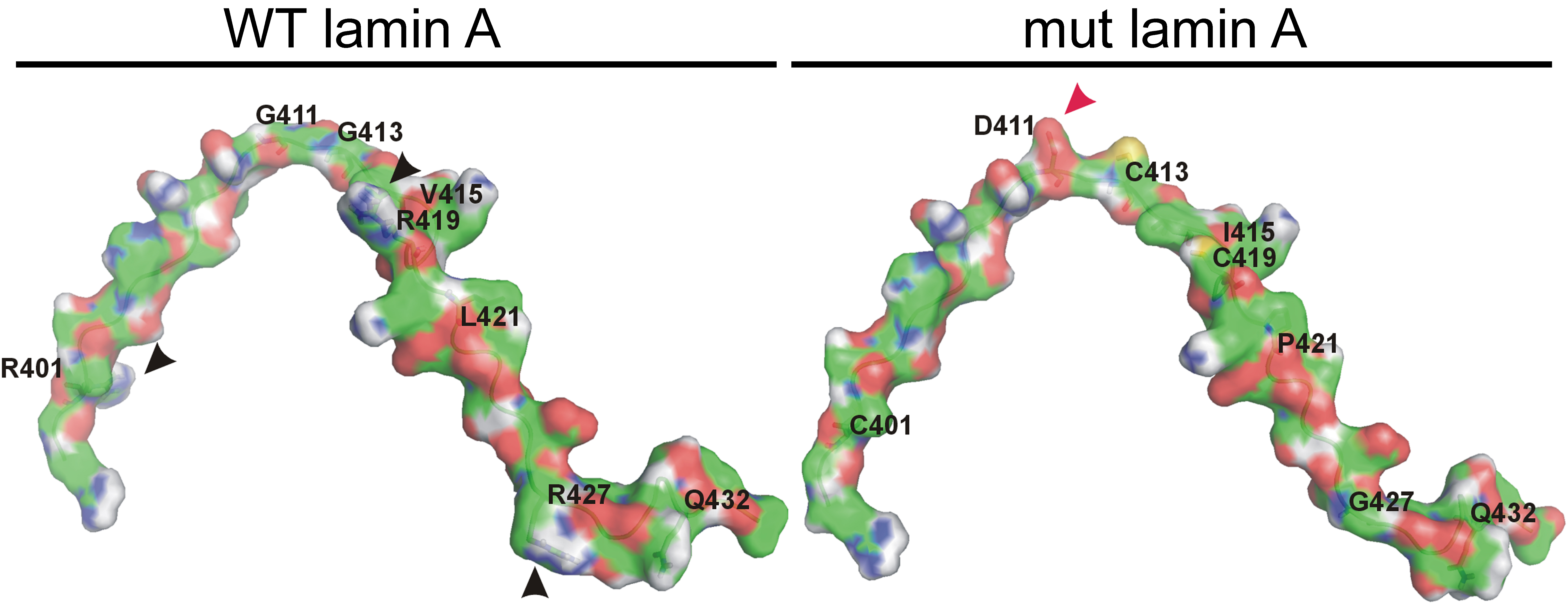

Supplement: Figure S4 — Molecular surface properties of WT lamin A and laminopathy causing lamin A mutantions. The figure shows a comparison of the surface rendering of WT lamin A aa 403–425 (left) and the same sequence including all lamin A mutations analysed here (right). Highly positive and negatively charged residues are shown in blue and red, respectively. Black arrowheads point on positively charged groups that are lost due to mutations. The red arrowhead points on a negatively charged group that is inserted by the lamin A mutation G411D. (TIF) [file pone.0071850.s004.tif]

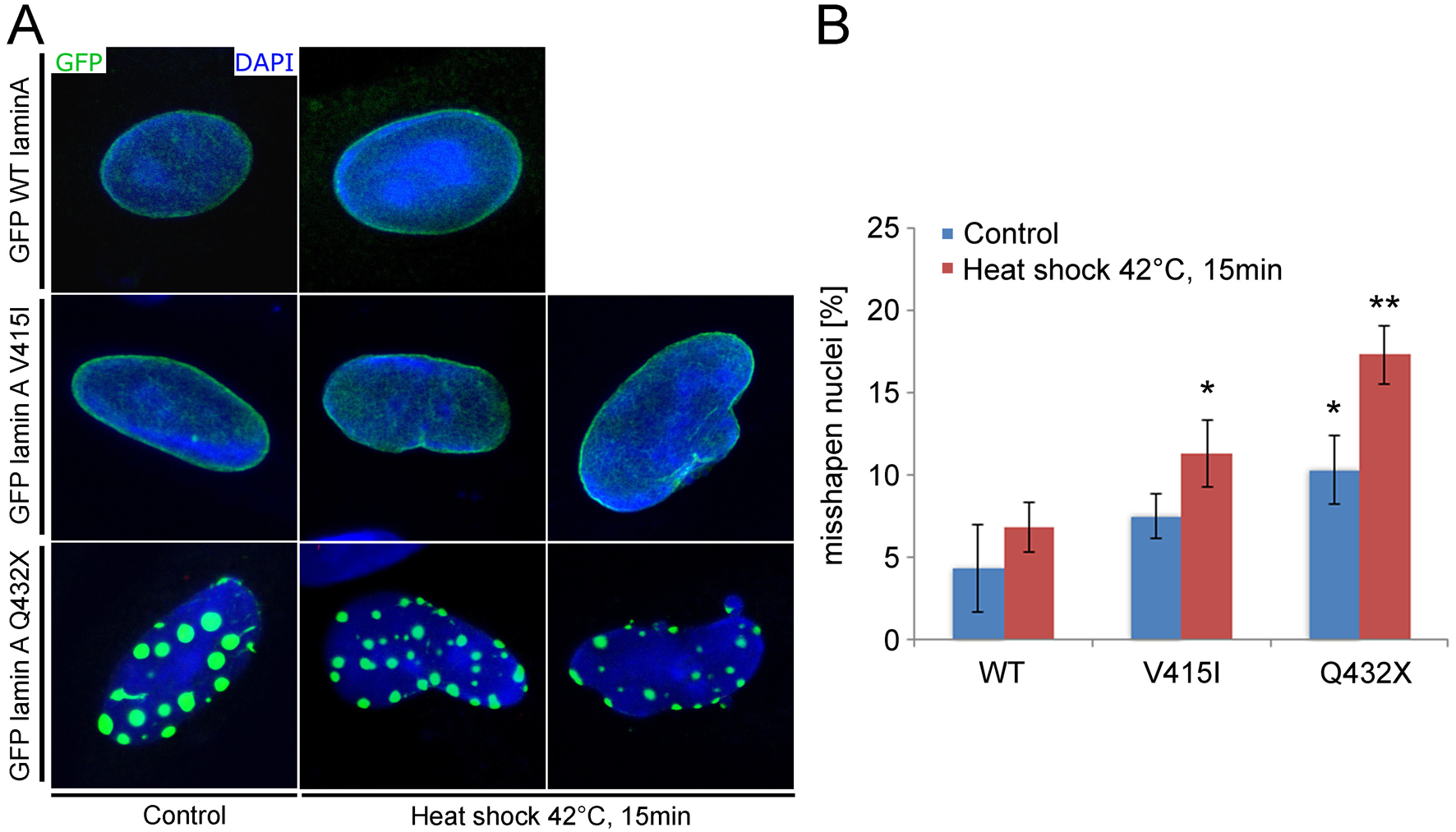

Supplement: Figure S5 — Lamin A mutations V415I and Q432X cause nuclear deformations in heat shock experiments. Human fibroblasts transiently expressing GFP WT, V415I or Q432X lamin A were exposed to a 15 minute heat shock at 42°C and fixed immediately to evaluate nuclear morphology by immunofluorescence (A) followed by and statistic analysis (B). Cells transiently expressing GFP lamin A WT were used as a reference. Two independent experiments were performed and 300 nuclei each were analysed. Nuclei from cells expressing GFP lamin A Q432X showed significantly higher amounts of deformations already before heat shock. P-Values of less than 0,01 are defined as significant (*) and below 0,001 as highly significant (**). (TIF) [file pone.0071850.s005.tif]
